# First Molecular Data and the Phylogenetic Position of the Millipede-Like Centipede *Edentistoma octosulcatum* Tömösváry, 1882 (Chilopoda: Scolopendromorpha: Scolopendridae)

**DOI:** 10.1371/journal.pone.0112461

**Published:** 2014-11-12

**Authors:** Varpu Vahtera, Gregory D. Edgecombe

**Affiliations:** 1 Zoological Museum, Department of Biology, University of Turku, Turku, Finland; 2 Finnish Museum of Natural History, Zoology Unit, University of Helsinki, Helsinki, Finland; 3 Department of Earth Sciences, The Natural History Museum, London, United Kingdom; Sars International Centre for Marine Molecular Biology, Norway

## Abstract

*Edentistoma octosulcatum* Tömösváry, 1882, is a rare, superficially millipede-like centipede known only from Borneo and the Philippines. It is unique within the order Scolopendromorpha for its slow gait, robust tergites, and highly modified gizzard and mandible morphology. Not much is known about the biology of the species but it has been speculated to be arboreal with a possibly vegetarian diet. Until now its phylogenetic position within the subfamily Otostigminae has been based only on morphological characters, being variably ranked as a monotypic tribe (Arrhabdotini) or classified with the Southeast Asian genus *Sterropristes* Attems, 1934. The first molecular data for *E. octosulcatum* sourced from a newly collected specimen from Sarawak were analysed with and without morphology. Parsimony analysis of 122 morphological characters together with two nuclear and two mitochondrial loci resolves *Edentistoma* as sister group to three Indo-Australian species of *Rhysida*, this clade in turn grouping with *Ethmostigmus*, whereas maximum likelihood and parsimony analyses of the molecular data on their own ally *Edentistoma* with species of *Otostigmus*. A position of *Edentistoma* within Otostigmini (rather than being its sister group as predicted by the Arrhabdotini hypothesis) is consistently retrieved under different analytical conditions, but support values within the subfamily remain low for most nodes. The species exhibits strong pushing behaviour, suggestive of burrowing habits. Evidence against a suggested vegetarian diet is provided by observation of *E. octosulcatum* feeding on millipedes in the genus *Trachelomegalus*.

## Introduction

The chilopod order Scolopendromorpha is a species-rich group composed of ca 700 species in 34 genera or subgenera classified in five families [1]. Although the relationships within the order have been recently clarified using molecular or combined molecular and morphological data [2]–[5], the placement of some rarely encountered taxa has still been made based on morphological data alone [6]–[9].

**Figure 1:**
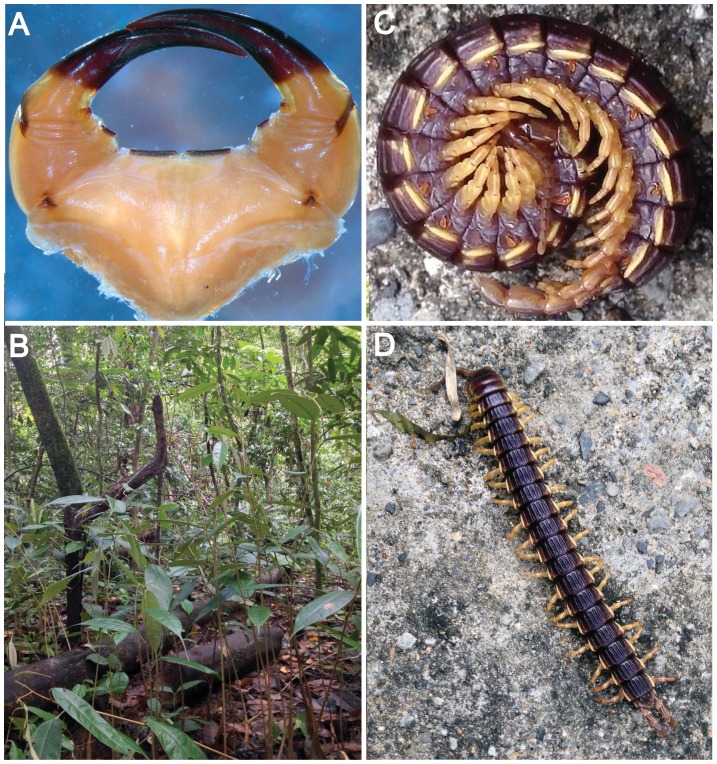
*Edentistoma octosulcatum* and its habitat. A, Forcipular segment in ventral view; B, alluvial forest where specimen was collected in March 2013; C–D, specimen used for DNA sequencing. C, walking; D, coiled.

While most species of the family Scolopendridae are fast and aggressive predators that may feed on prey even much larger in size than themselves, not all of them share this behaviour. Despite having diagnostic characters of the scolopendrid subfamily Otostigminae (e.g., round spiracles with the atrial floor raised in humps; lateral clusters of sensilla on the clypeal part of the epipharynx), the only member of the monotypic tribe Arrhabdotini Attems, 1930, *Edentistoma* Tömösváry, 1882 ( = *Arrhabdotus* Attems, 1930), differs in many respects from other members of the order. It moves slowly, is not aggressive, and superficially it could be confused with a millipede. Whereas the tergites in other scolopendromorphs are flexible, they are conspicuously rigid in *E. octosulcatum* species due to seven pronounced keels that span the tergites longitudinally. Despite bearing massive forcipules and housing a venom gland [6], *E. octosulcatum* has several adaptations that have been thought to indicate that its feeding habits may differ from those of other scolopendromorphs [10]. For example, it lacks tooth plates on the forcipular coxosternum (Fig 1A), typical structures of most scolopendromorphs (and other centipedes), which are thought to function as “can openers” when tearing the cuticle of arthropod prey [11]. In addition, the mandibular teeth of the species are small and the preoral chamber and mandibular lamina dentifera are densely covered in bristles [6]. Also, as shown by Koch et al. [7], the gizzard of the species is situated further anteriorly (in segment 5) than in other species of the order (segments 10–16) and unlike any other scolopendrid, it lacks the spinose armature on the gizzard plicae. Based on such adaptations, *E. octosulcatum* has been speculated to possibly feed by scraping cryptogams [10].

Due to its remote occurrence in Borneo and the Philippine island of Palawan (NHM London, leg. A. Everett; new record), *E. octosulcatum* is known from just a few museum specimens. The type specimen (collected by J. Xántus in Borneo in 1870) is kept at the Hungarian Natural History Museum, Budapest. There are no published reports of the species since it was last collected in 1978 during the Royal Geographical Society/Sarawak Government Expedition to Sarawak. During that expedition one individual was found walking on a small tree trunk in the *Kerangas* forest at night, prompting hypotheses that the species could be arboreal and restricted to this forest type [12].

### Previous hypotheses for phylogenetic position of *Edentistoma*

**Figure 2:**
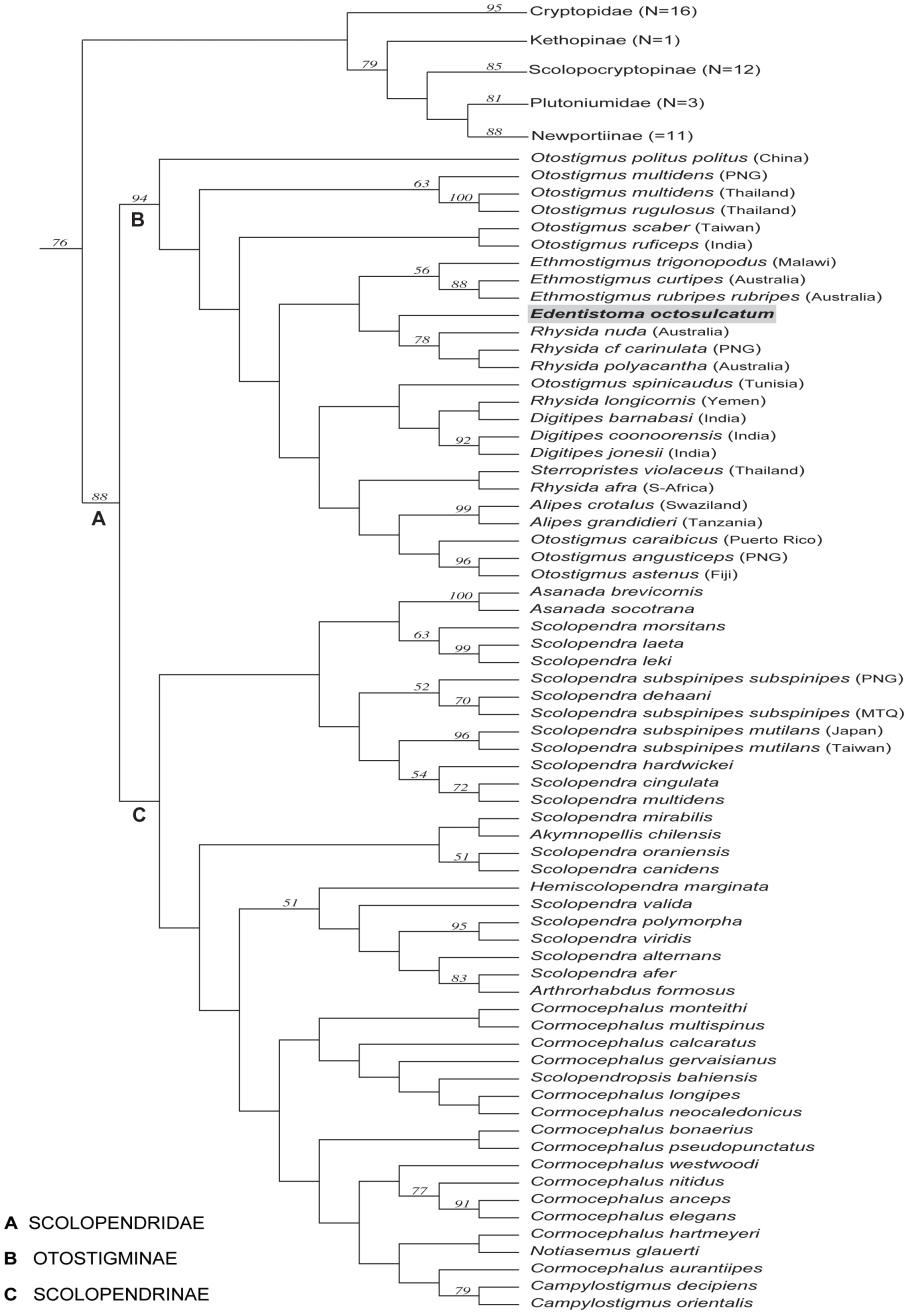
Optimal cladogram based on combined parsimony morphological and molecular data sets. Jackknife values >50 shown above the nodes. N = number of species.

**Figure 3:**
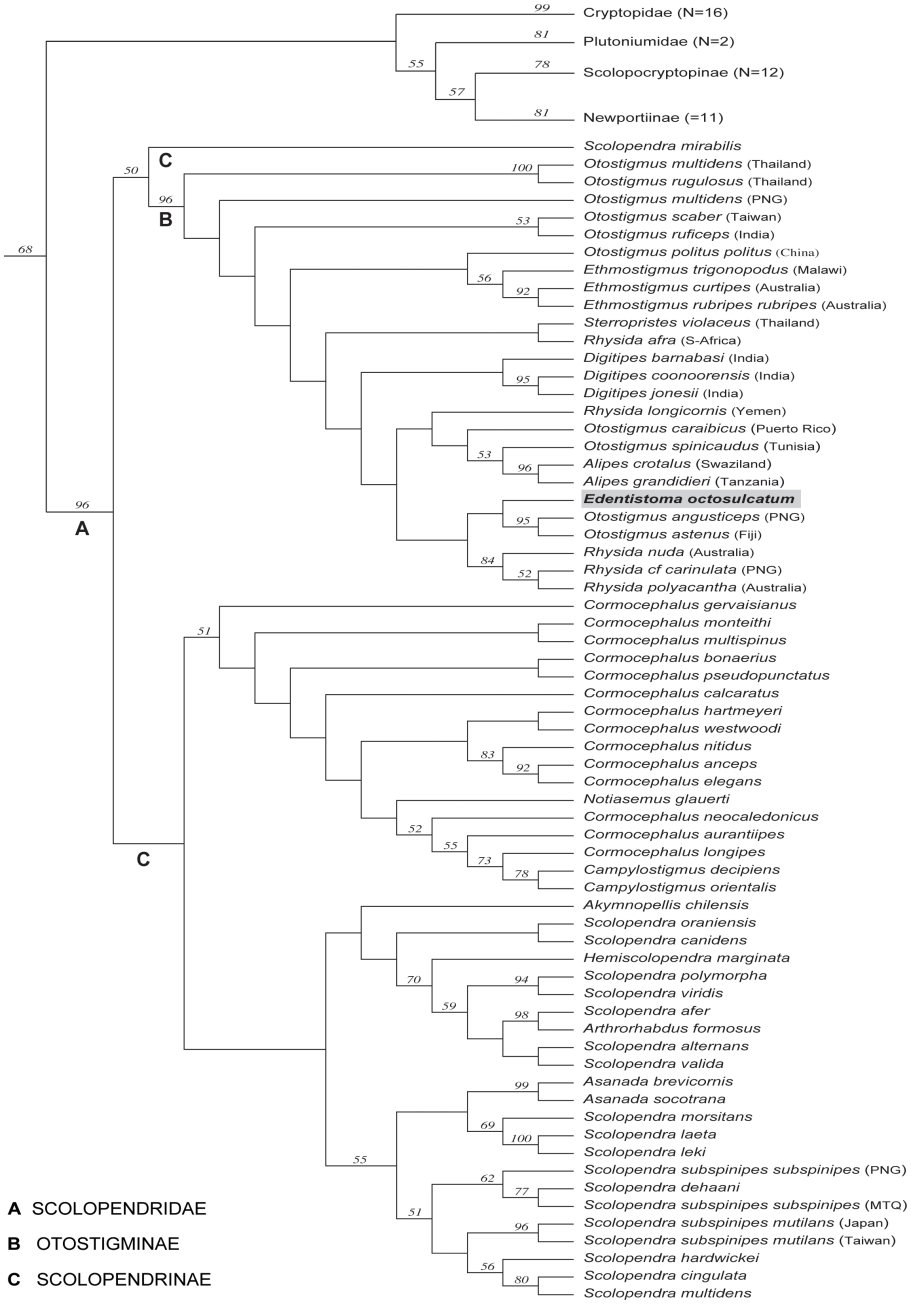
Optimal cladogram based on combined parsimony analysis of molecular data. Jackknife values >50 shown above the nodes. N = number of species.

To the present time, the phylogenetic position of *E. octosulcatum* has been studied using morphological characters only. However, these data alone have not been able to unambiguously resolve its position. Morphological analysis focusing on peristomatic characters resolved the species either as sister to Otostigmini or to Otostigmini + Scolopendrini [6], whereas the addition of gizzard characters [7] placed it at the base of the family Scolopendridae (excluding Asanadini). In the latest analyses of the order Scolopendromorpha [4], combined morphological (122 characters) and molecular (four markers) data were used to explore affinities among and within genera. *Edentistoma octosulcatum* was included in the analyses based on its morphological characters although no molecular data were available for it. The combined analysis of both data types placed *Edentistoma* within Otostigminae together with another peculiar southeast Asian genus, *Sterropristes* Attems, 1934 [see 13 for revision]. However, this sister group relationship was only weakly supported (jackknife frequency 63%). In the same paper (Figs 1–3) the 122-character morphological dataset was also analysed separately but the placement of *Edentistoma* changed depending on the concavity constant value used for implied character weighting (*k* = 2 grouped it inside of Otostigminae with *Ethmostigmus*, whereas *k* = 3 placed it in the base of the subfamily).

To more rigorously evaluate the phylogenetic position of *Edentistoma*, it was clear that molecular data of the genus needed to be included in the analysis. However, since the existing museum specimens were old, fresh material was required.

## Material and Methods

### Study site

Fieldwork was conducted in Gunung Mulu National Park, Sarawak, Malaysia in March-April 2013. Gunung Mulu is the largest national park in Sarawak, occupying an area of 544 km^2^ [14]. Chilopods were hand-collected over the course of 12 days, three nights and five days of which were spent at the same site where *E. octosulcatum* was found in 1978 during the Royal Geographical Society/Sarawak Government Expedition. The main forest types in this area are alluvial (lowland riverine), *Kerangas* and peat swamp forests.

Soil, fallen trees and tree trunks were searched both during the day and night. Simultaneously, hundreds of pitfall traps, situated in different forest types at different altitudes, were placed in the area by a research group led by I. Hanski. All chilopod material from those traps was given to the first author. A single specimen of *E. octosulcatum* was hand-collected on March 28, 2013 in an alluvial forest near Camp 5 (N 04°08.844′ E 114°53.384′) where it was found coiled under a log (Figs 1B–D). The specimen is deposited in the Zoological Museum, University of Turku, Finland (voucher ZMUT_Chi01). Field and export permits were granted by Forests Department, Sarawak.

### Molecular methods

DNA was extracted and four molecular markers (nuclear 18S rRNA, and 28S rRNA, mitochondrial 16S rRNA, and the mitochondrial protein-encoding cytochrome *c* oxidase subunit I (COI)) were amplified as described in Vahtera *et al*. [3], [4]. The new sequences are deposited in GenBank under accession numbers KM492928-KM492931. GenBank registrations for all other species analysed herein are provided in Vahtera *et al*. ([4], Table 1).

The morphological dataset coded as 122 characters ([4], Appendix 1) can be downloaded in nexus format as Morphobank Project P987, ‘Phylogenetics of scolopendromorph centipedes: Can denser taxon sampling improve an artificial classification?’ (http://www.morphobank.org/index.php/Projects/ProjectOverview/project_id/987).

### Phylogenetic analyses

#### Parsimony approach

The combined molecular and morphological datasets were analysed using direct optimization [15] as implemented in POY ver. 5.1.1 [16]. Analyses were conducted in parallel using 16 nodes in a high-performance supercluster Taito at CSC (IT-Center of Science), Finland.

Morphological data were analysed with equal character weights. The 18S rRNA and 16S rRNA fragments were treated as unaligned, meaning that sequences of different lengths were submitted to the analysis without aligning them beforehand. The COI and the two 28S rRNA fragments (amplicons *b* and *c*) were treated as prealigned. The 28S fragments were aligned with MUSCLE ver. 3.6 [17] and trimmed in Gblocks ver 0.91b [18], [19].

Fifteen hours of a timed search was conducted for both the combined morphological and molecular dataset and for the combined molecular data alone. The parameter set (3221: gap opening  = 3, gap extension  = 1, transversion  = 2, transition  = 2) that was found to minimize the incongruence between different data partitions in our earlier study was applied [4]. Nodal support was estimated using jackknife resampling [20] and the support values were mapped on the optimal tree.

#### Maximum likelihood approach

Multiple sequence alignments were first performed to each fragment using MUSCLE and the alignments were subsequently trimmed in Gblocks and concatenated using SequenceMatrix [21]. Maximum likelihood analysis was conducted using RaxML ver. 8.0.24 [22] in CIPRES Science Gateway v. 3.1 [23]. The likelihood analysis was performed conducting 100 independent searches applying a unique general time reversible (GTR) model of sequence evolution for each data partition with corrections for a discrete gamma distribution (GTR + Γ). Nodal support (1000 replicates) was estimated via the rapid bootstrap algorithm utilizing the GTR-CAT model [24].

## Results

### Behaviour

The specimen (Fig. 1D) was 60 mm long and moved slowly when compared to other scolopendromorphs (approx. 20 mm/s). Peculiar locomotory posture is seen when the specimen is observed from the side; it walks with the first segments arched, giving a hunchbacked impression (video S1). The specimen was not aggressive and instead of attacking it coiled on its side when touched (Fig. 1C, video S2). However, when placed inside a petri dish it first walked around the edges but then put its head towards the floor and pushed the first tergites strongly towards the lid, pushing the lid open. The specimen was strong and could easily open the lid even with some extra weight on top of it.

### Phylogenetic analyses: combined morphology & molecular data

The parsimony analysis resulted in a single optimal tree of 35,455 weighted steps (Fig. 2). In this tree, *E. octosulcatum* is resolved as sister group to three Indo-Australian *Rhysida* species (*R. nuda, R.* cf. *carinulata* and *R*. *polyacantha*). The closest relative of the *Edentistoma* + *Rhysida* clade is *Ethmostigmus* (*E. trigonopodus, E. curtipes, E. rubripes rubripes*). Although the support for membership of *Edentistoma* in Otostigmine is strong (jackknife frequency of 94 for the node that delimits the subfamily), the nodes that separate it from various clades (largely including species of *Otostigmus*) to the base of Otostigminae are all weakly supported. This is not surprising since nodal support throughout the subfamily Otostigminae is low.

### Phylogenetic analyses: combined molecular data

The single optimal tree (L = 34,947 weighted steps) resulting from the parsimony analysis of molecular data alone is shown in Fig. 3. Although monophyly of Otostigminae again receives strong resampling values (JF 96), the problem with low nodal support within the clade persists. In this analysis *Edentistoma* groups together with two *Otostigmus* species from the Pacific region, *O. angusticeps* and *O. astenus*. This clade in turn is resolved as sister to the same three Indo-Australian *Rhysida* species as were found in the analysis using combined morphological and molecular data sets.

**Figure 4:**
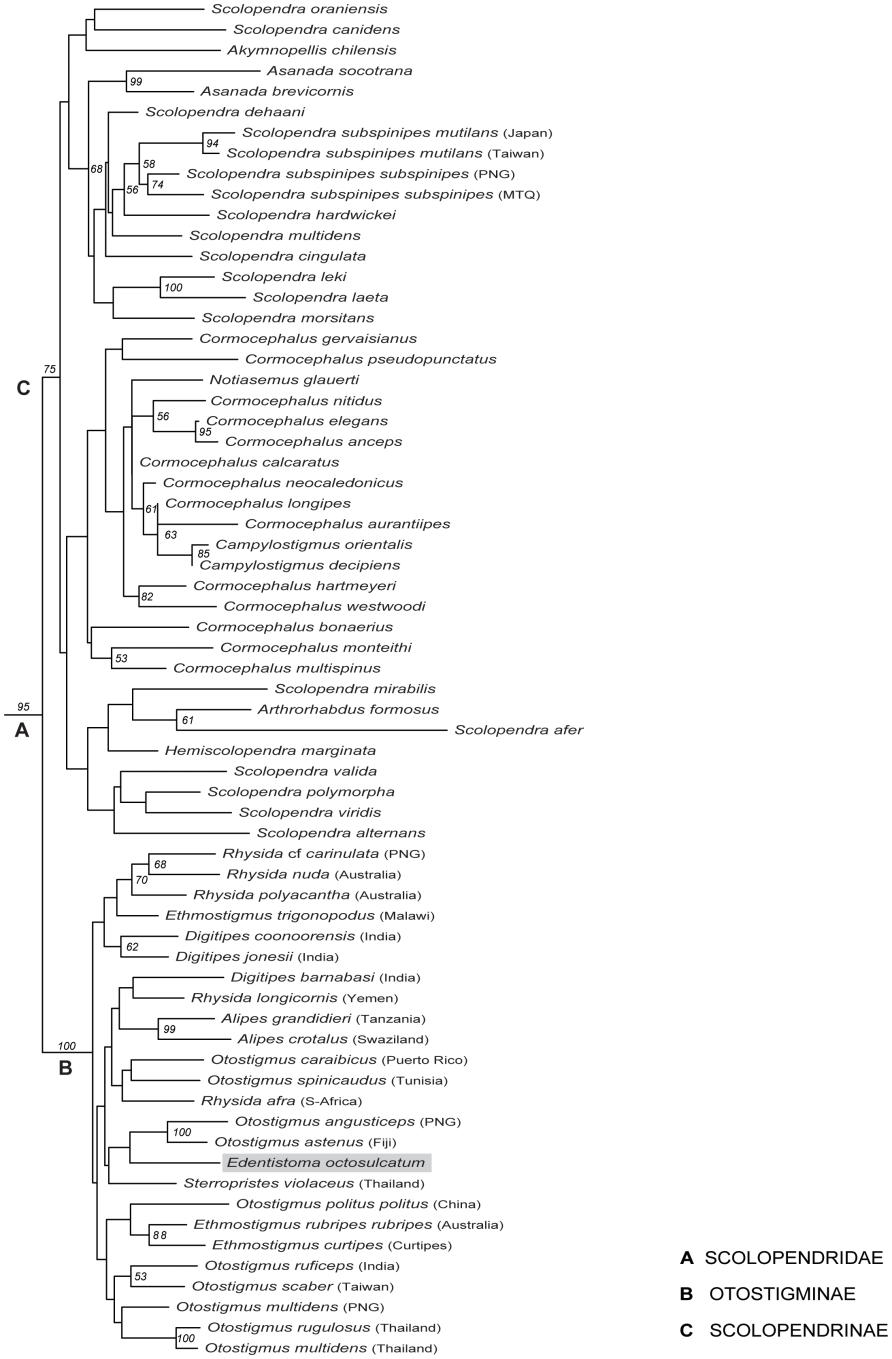
Optimal maximum likelihood tree based on combined molecular data. Bootstrap support values >50 on nodes.

The tree topology (Fig. 4) resulting from the maximum likelihood analysis (ln*L* = −40690.354189) is very much in line with the result of the combined molecular data alone analysed under parsimony. The likelihood result supports both the monophyly of Otostigminae (BS 100) and the placement of *Edentistoma* with *O. angusticeps* and *O. astenus*, although bootstrap values between the otostigmine taxa were mostly very low (BS<50). Again *Edentistoma* is deeply nested within Otostigmini.

## Discussion

The atypical walking posture (video S1), pushing behaviour and coiling when disturbed have not been reported from other scolopendromorphs. Pushing indicates the species may utilize burrowing or pushing in its environment (possibly in soil or fallen tree trunks).

The fact that the specimen was collected in alluvial forest demonstrates that it is not restricted to *Kerangas* forests. However, it does seem clear that *E. octosulcatum* is not abundant and is only rarely encountered. It may also be that its rarity is due to the lack of information about its lifestyle. If *Edentistoma* employs peculiar (to scolopendrids) behaviour, it may be that it avoids being collected when being searched using traditional collecting methods (under logs, bark and rocks).

With regards to feeding habits, predation on a specimen of the millipede genus *Trachelomegalus* has been observed. A photograph on the website http://www.creationearth.com/4326_photo_centipede_eating_milipededepicts *E. octosulcatum* holding a specimen of *Trachelomegulus*, having eaten the internal parts of the anterior body segments and discarded the exoskeleton, which was left as a set of disarticulated body rings. This observation of predatory behaviour is consistent with the typical development of a venom gland in *E. octosulcatum*.

### Status of Arrhabdotini and relationships within Otostigminae

Two competing schemes classify the tribe Arrhabdotini. The original one by Attems [25] treated it as a monotypic tribe including only a single species, *E. octosulcatum*. A different composition suggested by Schileyko [26], based mostly on the segmental distribution of spiracles, grouped *Edentistoma* and *Sterropristes* ( = *Malaccolabis* Verhoeff, 1937; see Muadsub *et al*. [13] for synonymy) in Arrhabdotini. In this view Arrhabdotini is part of Sterropristinae together with Ethmostigmini (*Alluropus*, *Ethmostigmus*, *Rhysida*). The cladistic analysis of morphological data by Schileyko and Pavlinov [27] found support for Arrhabdotini sensu Schileyko by resolving *Edentistoma* and *Sterropristes* as sister groups.

*Sterropristes* was not included in the subsequent morphological [6], [7], [28] or combined morphological and molecular [3] analyses of the order. The only analysis subsequent to that of Schileyko and Pavlinov [27] with both *Edentistoma* and *Sterropristes* included is by Vahtera et al. [4]. In this study, when morphological data were analysed alone under certain concavity constants for implied weights (*k* = 3), *Edentistoma* was resolved as the most basal otostigmine (consistent with its classification as a separate tribe), followed by *Sterropristes*. When both morphological and molecular data were analysed in combination, a weakly supported (JF 63) sister group relationship was found, although *Edentistoma* had only morphological data available.

Including the first molecular data for *Edentistoma* has now shown its closest evolutionary relatives vary depending on whether the analysis is based on both morphological and molecular evidence (Fig. 2) or on molecular data alone (Figs 3,4). Neither of the parsimony analyses (Figs 2,3) supports closest relationships with *Sterropristes*, but rather with species groups within *Rhysida* or *Otostigmus*. Although weakly supported, the likelihood analysis (Fig. 4) places *Sterropristes* basal to *Edentistoma*/*O. angusticeps* + *O. astenus* clade. Given the overall low levels of nodal support and the discordant placement of the two taxa between different analyses, a close relationship between *Sterropristes* and *Edentistoma* cannot be definitely discounted. Classification of *Edentistoma* on its own in the tribe Arrhabdotini is not supported by these data; *Edentistoma* consistently nests within Otostigmini rather than resolving as sister group to a monophyletic Otostigmini.

Regarding broader relationships within Otostigminae, the results of combined morphological and molecular data as well as the combined molecular data alone support the monophyly of *Ethmostigmus* using parsimony (JF 56 in both analyses) and *Alipes* (JF 99/96). The combined molecular analysis under parsimony (Fig. 3) provides some support for the monophyly of *Digitipes* (JF<50), though this is contradicted in the likelihood tree (Fig. 4). The clade formed by the Indo-Australian *Rhysida* species is consistently monophyletic (JF 78/84) whereas *R. afra* from South Africa and *R. longicornis* from Socotra (Yemen) never group with these other three congeners or with each other. *Otostigmus* is likewise polyphyletic in all three analyses but can be seen to comprise three groupings that have biogeographic signal: a basal grade is composed of Asian and Pacific species, whereas the exemplars from north Africa and the Canary Islands (*O. spinicaudus*) and the Caribbean (*O. caraibicus*) are allied to other otostigmine genera.

## Conclusions

*Edentistoma* is placed in different parts of Otostigminae/Otostigmini depending on whether the analysis is based on morphological or molecular data only or on the combined analysis of them both. Since low nodal support is found throughout the subfamily, irrespective of the optimality criterion used (parsimony or likelihood), the problem appears to touch the whole clade instead of affecting *Edentistoma* in particular. The standard markers used in this study adequately resolve relationships within other studied scolopendromorph clades (i.e. Cryptopidae, Scolopocryptopidae), so we surmise that the problem might be overcome with more comprehensive taxon sampling. With its currently known 115 valid species [29], Otostigminae is one of the most species-rich scolopendromorph subfamilies. Our current analysis included 25 of these species, thus leaving out almost 4/5 of the known species. This gap in taxonomic sampling might contribute to weak nodal support.

## Supporting Information

Video S1 **Peculiar locomotory posture of** ***E. octosulcatum*** **.**
(ZIP)

Video S2 ***E. octosulcatum*** **coiling when disturbed.**
(ZIP)
